# Supplementary material for: Socioeconomic inequalities in the risk factors of noncommunicable diseases (hypertension and diabetes) among Bangladeshi population: Evidence based on population level data analysis
Source: PLoS One. 2022 Sep 20;17(9):e0274978. doi: 10.1371/journal.pone.0274978 (PMC9488802; doi:10.1371/journal.pone.0274978)
Supplement: Attachment — Submitted filename: Rebuttal.docx [file pone.0274978.s001.docx]

**Author’s response to reviewer’s comments.**

PONE-D-22-09701

Socioeconomic Inequalities in the Risk Factors of Noncommunicable diseases (Hypertension and Diabetes) Among Bangladeshi Population: Evidence based on population level data analysis

I thank the editor and reviewers for their comments and appreciate the care taken in reviewing our manuscript. I hope that my response provided below adequately addresses the concerns of the respective reviewers. I have also taken the opportunity to correct any outstanding grammatical errors.

|  | **Reviewer’s comments** | **Author’s Responses** |
| --- | --- | --- |
| **Reviewer #1** |  |  |
|  | 1. Did the author exclude hypertension cases with pregnancy? Because hypertension cases may resolve after childbirth. | I excluded pregnant women for the analysis. Page-4, Line 129 |
|  | 2. Author may look at individual association between risk factors for hypertension and diabetes. | Table 3, shows the individual association between the risk factors for hypertension and diabetes. |
|  | 3. Some studies have showed higher hypertension rate in working people since most of current jobs are desk jobs. Author found in this study that people with jobs have decreased risk of hypertension and diabetes. This is an interesting area to explore, e.g. job nature, working hour. If not explored, dissimilarities like this can be included in the discussion section. | Thanks for noting this. The main intention of this research was to measure the inequality of hypertension and diabetes and the factors associated with the inequality therefore I deter discussing the regression results in the main Discussion section. However, further study is warranted to validate this finding why occupational involvement is associated to lower the risk of hypertension and diabetes. |
|  | 4. The discussion section of the article is not well organized. The primary focus of the research question is 'socioeconomic inequality'. Therefore, while writing the discussion section, author may organize it by socioeconomic factors. | I have organized the discussion based on the findings of Table 4, the decomposing results. Here, the separation of socioeconomic factors is not necessary because my intention was to measure the factors associated with the inequality. And to measure the inequality “socioeconomic factor” “wealth status” was considered. This is the appropriate statistical method to measure the inequality in health research. |
|  | 5. Author may link the study finding with literature more rigorously. | Thanks for noting this, I have tried my best to validate the findings with the most recent literature found in the PubMed and GoogleScholar. However, I have faced shortages of paper because there are not enough research papers which have been measured “socioeconomic inequality” of both hypertension and diabetes”. |
|  | 6. The website provided in the data availability section (<https://dhsprogram.com/>) do not share data with third party without permission. Hence, it is advisable to make the dataset for diabetes and hypertension visible / available to the editor for credibility of the analysis. | Thanks for your suggestion, DHS dataset is available upon request, any third-party users cannot share it by its policy obligations. The DHS dataset used in the study is reliable and trusted. In addition, I may assure you that similar study and findings are unavailable in the internet. Because doing the study I have searched in PubMed, GoogleScholar. |
|  |  |  |
| **Reviewer #2** | I must appreciate you for writing a nice manuscript focusing on NCDs including Diabetes and Hypertension.  However, I have some suggestions regarding your manuscript from the point of view of a reviewer.  Abstract: Nice, simple, and highly expressible.  Background: Excellent  Methodology: Nicely discussed and statistically sound. | Thanks for your appreciation and kind words. |
|  | Results: The tables and figures were well interpreted. Some important variables (added salt, working environment, food habit, family history of NCDs, lifestyle, smoking, alcohol, tobacco, etc. ) regarding NCDs are missing here, however, these limitations are manageable during working with the secondary dataset. I would like to add here that there is huge room to improve in the linguistic section, especially in sentence making, parts of speech, and run-on sentences. | I Agree with you that the following variables are important for developing hypertension and diabetes. But the original dataset of DHS doesn’t have the necessary information which has been described in the limitation section. Page 20, Line 461-463. I have re-edit the result section for linguistic anomalies. |
|  | Discussion: The article was nicely discussed and compared with the other related published articles. I think that linguistic improvement is also necessary for this section. I shall hereby suggest taking help from the professional body. | Thanks for your appreciation and kind words I have checked the entire paper and tried my best to make it errors free. |
|  | Conclusion: Briefly, the entire article is reflected in this section. | Thank you very much. |
|  | I must be thanking you once again for preparing this manuscript and have good luck with your manuscript. | Thanks for your acknowledgment and appreciation. |
|  |  |  |
| **Reviewer #3:** | 1. The paper is not well written. Some sentences are long with limited references. The justification of the study is poorly written there is necessity and room for improvement. | Necessary corrections have been made. Some sentences have been broken down and insert some new references. However, rest of the three reviewers said this is a well-written manuscript therefore, I didn’t change much.  The justification/rationality of the study clearly discussed in the background section. Page 3-4, Lines 102-114. |
|  | 2. Methods section: 1. Data source is described to be the DHS. A little more specific details of how the data were obtained, what procedures were followed and long with what, if, a letter was required and for which datasets/variables data was accessed and how long it took would give readers more picture of the DHS data access. | The data source and sampling design have been clearly stated in the methods sections. To collect the DHS data we need not letter, just need to create an account and application in what purpose you want to use the dataset. Their details procedures have been discussed in the BDHS 2017-18 report along with their respective website. |
|  | 3.Results: Informative a bit lengthy. Also could use language and editorial revisions. some of the sentences were too long and difficult to understand the intent. | I don’t think the results section is bit lengthy and confusing. I have revised and re-read the whole section. |
|  | 4.Conclusion: Nothing new is found requires major revision, appears too small. Suggest some recommendations. | I disagree with your statement. Please follow reviewer #2 comments regarding the conclusion.  I have tried by best to reflect the findings simply in the conclusion.  Since this study used cross-sectional dataset therefore, I deter suggesting recommendations, because most of the reviewers suggest not doing recommendations in cross-sectional study. |
|  |  |  |
| **Reviewer #4:** | Dear authors,  Thank you for the opportunity to review your manuscript. I found the paper to be generally well written, and the topic to be interesting. My comments/concerns with the current manuscript are noted below. | Thanks for your appreciation and found it interesting. |
|  | Methods: 1. Study population and survey design – what were the inclusion criteria used in order to arrive at the 12,290-sample size? Also, please clarify whether this represents the weighted or unweighted sample size. | Inclusion criteria: those who have valid BP and fasting plasma glucose and BMI and not pregnant were considered for analysis.  The total sample is weighted. The svy command was used to weight the dataset as recommended by DHS platform. |
|  | 2. Outcome variables – what was the reason for merging the patients with prediabetes and those with diabetes? Please clarify this within the text. | Thanks for noting this out. I have rephrased the sentence. |
|  | 3. (Major) Independent variables (page 4, first sentence) – the first sentence is missing a citation. Also, more details on the variables included are needed as they are of considerable importance to the interpretation of later findings. | References inserted.  Why the variables are of importance and its contribution to develop the inequality have been described in the discussion section and validated with appropriate referencing. |
|  | a. I encourage you to consider adding a conceptual model section that clearly outlines the categories of variables that influence your outcome variables, and then to list/explain the variables you include in order to adjust for each of these effects/channels within the context of your study. | I must be thanking of you for raising an important issue to draw a conceptual model. In this research paper two outcomes’ variables were considered i.e. hypertension and diabetes and both two effects each other. It was possible to draw if I only use one outcome variable and regression model. But in this paper my prime intention was to identify the factors which contribute to the inequality of hypertension and diabetes not the risk factors associated with developing hypertension and diabetes. In this point of view, I think you will consider not drawing conceptual framework/model for this study. |
|  | b. Page 5: why are you including age covariates among your independent variables? I ask this because the abstract and statistical analysis section notes that your outcomes are already age-standardized. | Age has direct impact on contributing hypertension and diabetes which is proved by published literature. I use age-standardization only when showed the prevalence therefore, there is no issue when I use regression or decomposition model. Same study is available in online <https://doi.org/10.1016/j.deman.2021.100012> |
|  | c. Are the “wealth status” categories based on pre-set cutoffs, or upon wealth deciles that you create form your data? Please clarify this within the text. | Thanks for pointing this out. I have used pre-set cutoffs. It is already measured therefore; no further measurements were required. |
|  | 4. Statistical Analysis: a. How was age standardization of the data done? Please consider adding details to an appendix. | This is just a statistical command which is available in the online. |
|  | b. Page 7: the reference numberings do not align with those within the reference section at the end, please correct this. | I have checked the references in the mentioned Page 7, all four references have been inserted in the reference section. |
|  | c. Page 7: the term elasticity is mentioned, but not defined. Please add definition and explanation of this measure. | It has now been discussed. Page 7-8, Lines 244-255. |
|  | 10. Results/Discussion: in presenting the main findings as percentages, it is important to: (a) provide additional details within the text on how these are computed within Table 4; and (b) clarify to readers that these do not necessarily sum to 100%. While this might be clear to practitioners of these methods, given the broad readership of PLOS One, I think it is important to explain this within the text to avoid confusion. | Thank you for your observations.  I think the discussion and the results section clearly stated your observations. In addition, Page 7-8, Lines 244-255, Page 15, Lines 355-356, will help the readers to know about the particular results. |
|  | Minor Comments: Background: 11. Page 2, paragraph 1: the last sentence is unclear and needs to be revised. | It has been revised. Now it reads “Therefore, addressing the health inequalities is important and it is gaining policy recognitions globally including developing country like Bangladesh.” |
|  | Methods: 12. Statistical Analysis – the sentence starting with “Then, we assessed hypertension’s age… “ is incomplete. Please revise. | Sorry for the unwanted mistake, it has now been corrected. |
|  | Results: 13. All tables – please add footnotes with explanation of all abbreviations under the table. | Corrected as suggested. |
|  | 14. Discussion – page 19, paragraph 2, first sentence: please clarify what is meant by “…significantly lower among the less socioeconomic conditions…”. | Sorry for the mistake, it has been corrected. |
