# Supplementary material for: Socioeconomic inequalities in the risk factors of noncommunicable diseases (hypertension and diabetes) among Bangladeshi population: Evidence based on population level data analysis
Source: PLoS One. 2022 Sep 20;17(9):e0274978. doi: 10.1371/journal.pone.0274978 (PMC9488802; doi:10.1371/journal.pone.0274978)
Supplement: Attachment — Submitted filename: Rebuttal.docx [file pone.0274978.s002.docx]

**Author’s response to reviewer’s comments**

PONE-D-22-09701R1

Socioeconomic Inequalities in the Risk Factors of Noncommunicable diseases (Hypertension and Diabetes) Among Bangladeshi Population: Evidence based on population level data analysis

I thank the editor and reviewers for their comments and appreciate the care taken in reviewing our manuscript. I hope that my response provided below adequately addresses the concerns of the respective reviewers. I have also taken the opportunity to correct any outstanding grammatical errors.

| Reviewer 4's | comments have not been adequately addressed. Specifically, we would like to see the following elements more carefully included in the manuscript: | Sorry to make you disappoint although I had tried my best addressing all your previous comments. I hope this version would satisfy you. |
| --- | --- | --- |
|  | (1) additional details on certain data / measures / definitions are needed throughout; | Thanks for your comments. In this study two outcome variables were used Hypertension and Diabetes. The details definition and measurement have been described under Outcome variables section.  Line 134-143 |
|  | (2) the final (main) decomposition analyses depend on the variables included within the model, and as such, it seems particularly important for the authors to explain/motivate their choice of variables beyond what is currently done (it would additionally be beneficial for them to base these variable choices on a clear conceptual model/framework, somethings that is currently missing); | Thanks for your comments. New conceptual framework has been inserted. Page-5 |
|  | (3) the interpretations of results are presented as percentages off of the overall CIX value, which needs to be clearly explained in order to avoid confusing many readers who might, for example, assume these sum to 100% (which they do not). | Thanks for your comments. Following the lines clearly described the interpretations of the results.  Line 234-245  Line 325-346 |
| Editor’s comments | - Please note that while reviewers commented on the manuscript being well written, the grammatical errors that currently exist throughout this manuscript have to be addressed before it can be considered for publication. In addition, please remove the first person tense. We invite you to visit the PLOS Writing Center for free resources found here: <https://plos.org/resources/writing-center/?utm_medium=website&utm_source=journalsite&utm_campaign=writingcenter&utm_content=submissionguidelines> | All outstanding grammatical errors have been solved, and all first-person tense have been removed. |
|  | - Additional specific edits noted by the editor: 1) Patient centered language is needed. For example, Line 102 “80% of the diabetic people reside” is not appropriate. See <https://doi.org/10.2337/dci17-0041> The Use of Language in Diabetes Care and Education. 2) Line 106, please provide the date of the recent nationwide survey with some explanation as to why there would be such a significant rise in prevelance. For example, does the methodology vary and therefore did not adequately capture prevalence? 3) Line 162, please use phrasing consistent with the dataset description for diabetes oral medications and site reference. 4)Line 174, grammar. Had no working…this description appears to be incomplete. | 1. Diabetic have been converted into diabetes throughout the paper. 2. Why the prevalence increased possible reasons have been described with appropriate references. Line 94-99 3. The BDHS 2017-18 report summery used “Prescribed Medicine” Page-BDHS 219)” therefore, I used prescribed medicine. Line 148 4. Sorry for the unwanted mistake. It has been corrected now it reads (Had no work) |
