# Supplementary material for: Socioeconomic inequalities in the risk factors of noncommunicable diseases (hypertension and diabetes) among Bangladeshi population: Evidence based on population level data analysis
Source: PLoS One. 2022 Sep 20;17(9):e0274978. doi: 10.1371/journal.pone.0274978 (PMC9488802; doi:10.1371/journal.pone.0274978)
Supplement: Attachment — Submitted filename: Rebuttal.docx [file pone.0274978.s003.docx]

**Author’s response to reviewer’s comments**

PONE-D-22-09701R2

Socioeconomic Inequalities in the Risk Factors of Noncommunicable Diseases (Hypertension and Diabetes) among Bangladeshi Population: Evidence Based on Population Level Data Analysis

I thank the editor for her very specific comments and corrections made in the paper rigorously, and I appreciate the care taken in reviewing my manuscript. I hope that my response provided below adequately addresses the concerns.

|  | Please review your reference list to ensure that it is complete and correct. If you have cited papers that have been retracted, please include the rationale for doing so in the manuscript text, or remove these references and replace them with relevant current references. Any changes to the reference list should be mentioned in the rebuttal letter that accompanies your revised manuscript. If you need to cite a retracted article, indicate the article’s retracted status in the References list and also include a citation and full reference for the retraction notice. | I have checked all the references all are corrects and no retracted papers have been cited. |
| --- | --- | --- |
|  | Introduction:  Line 93  Please rephrase to “…80% of people living with diabetes reside in…” | Rephrased the sentence as suggested. Line 87-90 |
|  | Line 99  Please rephrase to “Due to demographic transitions and….” | Corrected as suggested. |
|  | Methods:  Line 122 - BDHS spell out initially  Line 155 – Change “considered diabetes” to “….were classified as having diabetes” | Done as suggested. |
|  | Statistical Analysis  Line 189-191  Please change this statement to read:  “As a result, the prevalence of hypertension and diabetes were normalized in the same standard population in order to eliminate or reduce the effect of participant age and sex distribution discrepancies.” | Corrected as suggested. |
|  | Line 193-194  Please change this statement to this statement: “Following that, a log-binomial regression model with survey weights and explanatory variables with p-values (0.05) was used…” | Solved as suggested. |
|  | Line 224  Please add “the”: Among the population… | Corrected as suggested. |
|  | Results  Line 280  Please rephrase to: “A total of 12,290 were included in the final analysis.”  Line 288  Please do not use the word objects to refer to human subjects. Please rephrase to “…among the sample population” | Done as suggested. |
|  | Line 290 refers to “women” the preceding sections refer to “females”. For consistency please refer to all as “women” throughout the manuscript. | Thanks for your comment |
|  | Line 307 please correct, current sentence states “log binomial passion” | Thanks for your correction. Corrected as suggested. |
|  | Line 365 refers to diabetes and prediabetes. The manuscript as a whole is focused on diabetes and hypertension. Please address if prediabetes is also being examined. | Sorry of the unwanted mistake. |
|  | Line 435 please remove “to” from this sentence so that it reads, “may be responsible…” | Solved as suggested. |
|  | Line 437 please add “are” so that this sentence reads “…hypertension and diabetes are significantly lower…” | Corrected as suggested. |
|  | Line 443-444 please address grammar by rephrasing to: “While overweight and obesity are risk factors for hypertension and diabetes…” | Thanks. solved as suggested. |
|  | Line 471-472 please address grammar by rephrasing to: “Another factor contributing to overweight and obese…” | Thanks for your correction. Corrected as suggested. |
|  | Line 492, pattern is used twice. Please remove duplicate. | Sorry for the mistake, solved as suggested. |
|  | Line 510, please address, is this referring to plural studies or one study. If plural, please state “Previous studies have tried...” If singular, please state, “A previous study tried…” please add references to site which prior studies have tried. | Thanks for your comment, this line has been expunged. |
|  | Line 528, please address grammar by stating: “The present study concludes….” | Sorry for the mistake, it has been now corrected. |
